# Supplementary material for: A Cell-Autonomous Cold Sensor Promotes Lipolysis Through Phase Separation
Source: bioRxiv. 2026 Oct 27:2026.10.22.760470. Preprint. [Version 1] doi: 10.64898/2026.10.22.760470 (PMC13733734; doi:10.64898/2026.10.22.760470)
Supplement: Supplementary file 1 [file NIHPP2026.10.22.760470v1-supplement-1.pdf]

bioRxiv preprint doi: https://doi.org/10.64898/2026.10.22.760470; this version posted October 27, 2026. The copyright holder for this preprint (which was not certified by peer review) is the author/funder, who has granted bioRxiv a license to display the preprint in perpetuity. It is made available under a CC-BY 4.0 International license.

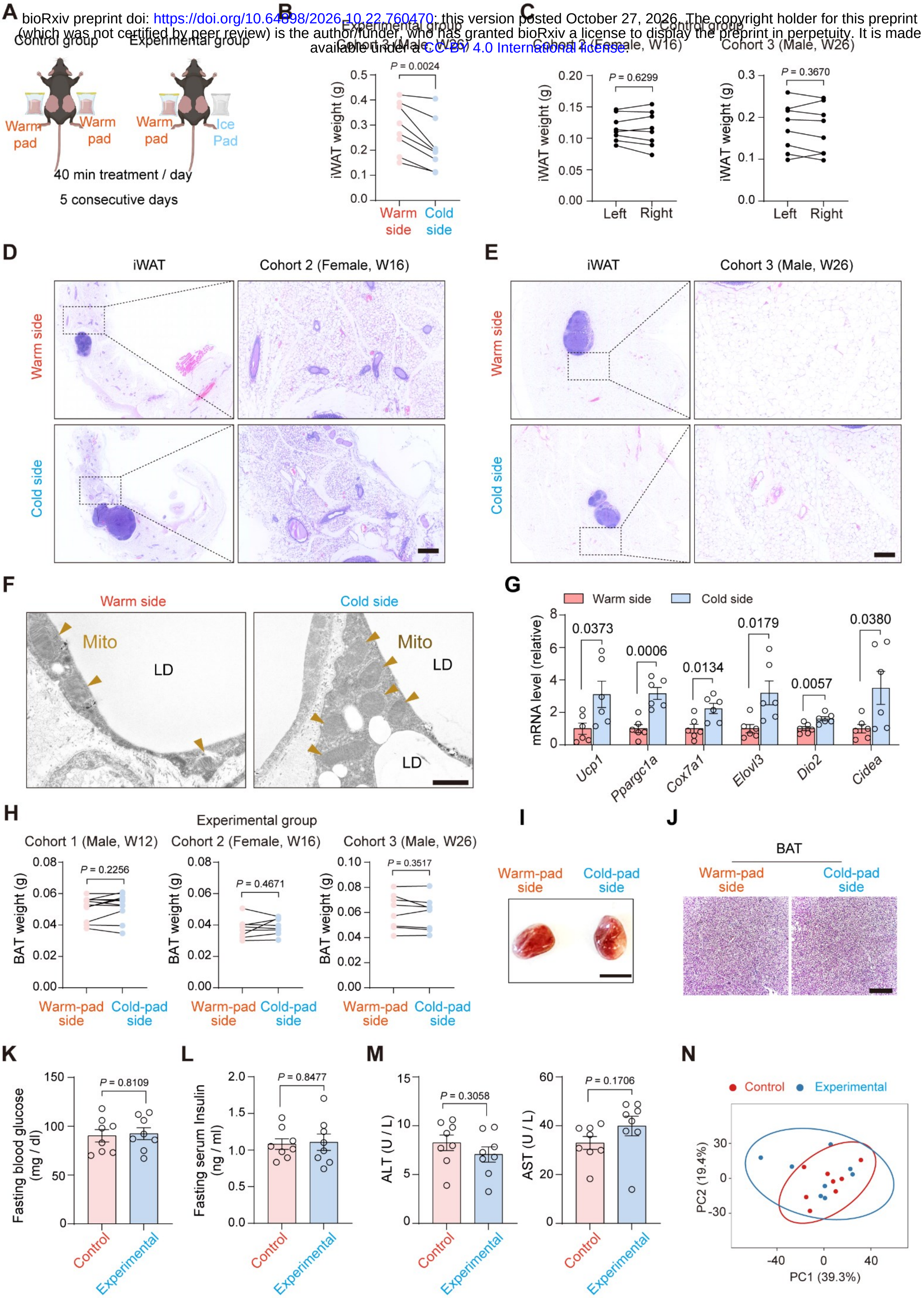

bioRxiv preprint doi: https://doi.org/10.64898/2026.10.22.760470; this version posted October 27, 2026. The copyright holder for this preprint (which was not certified by peer review) is the author/funder, who has granted bioRxiv a license to display the preprint in perpetuity. It is made available under a CC-BY 4.0 International license.

Supplementary Figure 1. The validation of tissue cooling effect on adipose tissue mass and systemic metabolic parameters.

A. Schematic illustration of the local adipose tissue cooling experiments. Mice housed at room temperature were shaved on both inguinal sides. Under anesthesia, the control group (Left) was placed on warm pads on both inguinal sides, whereas the experimental group (Right) was placed on a warm pad on one inguinal side and an ice pad on the other side for 10 min. This procedure was repeated 4 times per day, resulting in a total of 40 min of local cold exposure per day for 5 consecutive days.

B. iWAT weight of Cohort 3 (26-week-old male mice) following 5-day treatment with ice/warm pad. Each dot represents one depot, with matched warm- and cold-treated depots from the same mouse connected by lines. n = 8. Paired t-test.

C. iWAT weight of the control group mice that received warm pads on both inguinal sides. Each dot represents one depot, with matched depots from the same mouse connected by lines. Left, cohort 2 (16-week-old female mice); Right, cohort 3 (26-week-old male mice). n = 8 mice per cohort. Paired t-test.

D. Representative H&E staining of iWAT sections from cohort 2 that received either a warm pad or an ice pad. Left, whole-slide scans; Right, magnified views of the indicated regions. Scale bar: 200 µm.

E. Representative H&E staining of iWAT sections from cohort 3 that received either a warm pad or an ice pad. Left, whole-slide scans; Right, magnified views of the indicated regions. Scale bar: 200 µm.

F. Representative transmission electron microscopy images of iWAT that received either a warm pad or an ice pad. Arrowheads indicate mitochondria. Scale bar: 1 µm.

G. mRNA levels of thermogenic genes in iWAT from mice that received local cold-pad or warm-pad treatment for 5 days. Gene-expression levels were normalized to the warm-pad control group. n = 6 mice per group. Data are mean ± s.e.m. Unpaired t-test.

H. Interscapular BAT weight of three independent cohorts that received warm pads on both inguinal sides. Each dot represents one BAT lobe, with matched warm- and cold-treated depots from the same mouse connected by lines. Cohort 1, 12-week-old male mice (n = 11); Cohort 2, 16-week-old female mice (n = 8); Cohort 3, 26-week-old male mice (n = 8). Paired t-test.

I. Representative tissue images of interscapular BAT depots from mice in (**H**). Scale bar, 0.5 cm.

J. Representative hematoxylin and eosin (H&E) staining of BAT sections in (**H**). Scale bar, 210 µm.

K. Fasting blood glucose levels in cohort 3 mice. The control group (both warm pads) and the experimental group (one ice pad and one warm pad) were fasted for 6 h before measurement. n = 8. Unpaired t-test.

L. Fasting serum insulin levels in cohort 3 mice. The control and experimental groups were fasted for 6 h before measurement. n = 8. Unpaired t-test.

M. Serum alanine aminotransferase (ALT; left) and aspartate aminotransferase (AST; right) levels in cohort 2 mice. n = 8. Unpaired t-test.

N. Principal component analysis (PCA) of global serum lipid species in cohort 2 mice. n = 8.

bioRxiv preprint doi: https://doi.org/10.64898/2026.10.22.760470; this version posted October 27, 2026. The copyright holder for this preprint (which was not certified by peer review) is the author/funder, who has granted bioRxiv a license to display the preprint in perpetuity. It is made available under a CC-BY 4.0 International license.

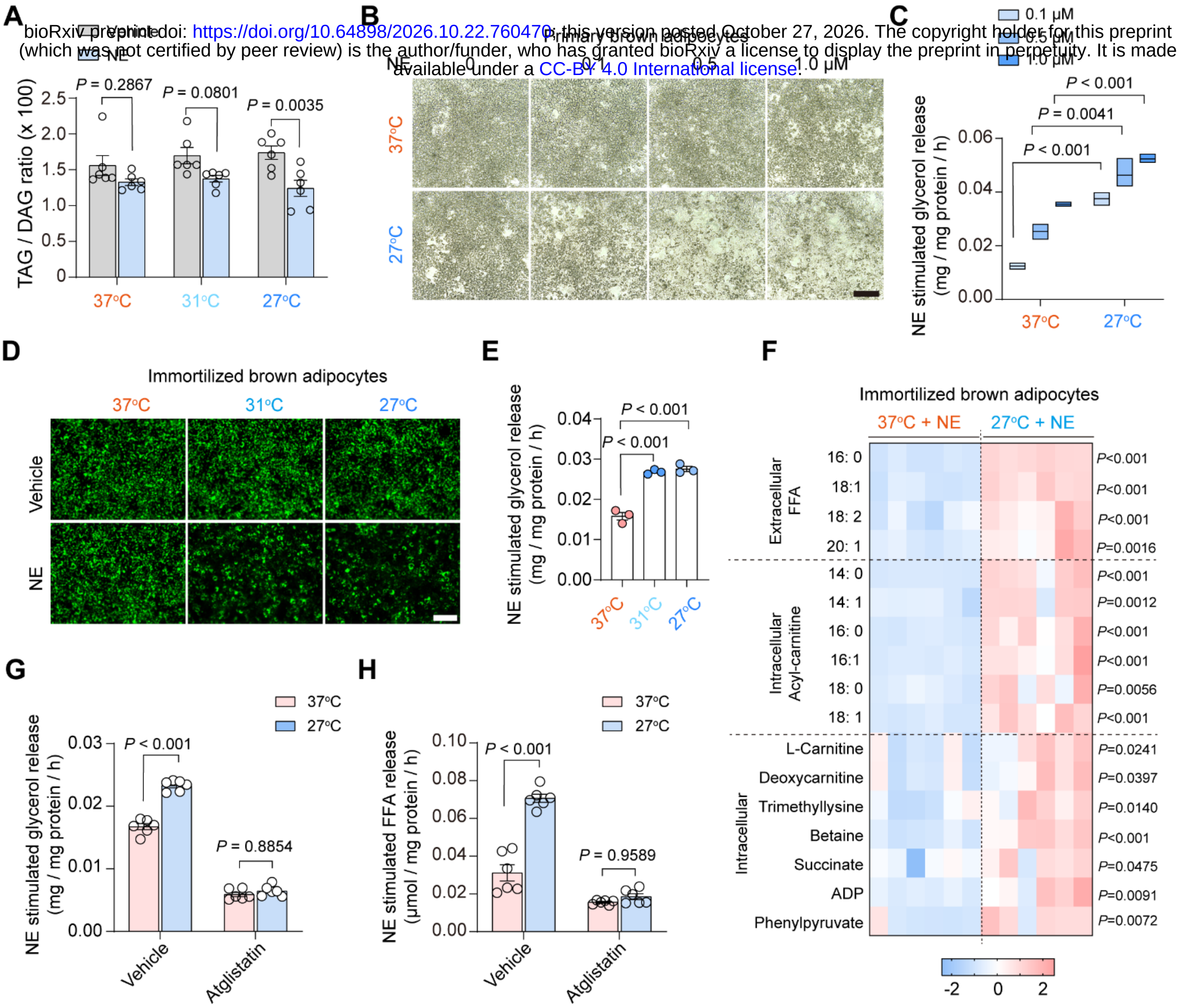

Supplementary Figure 2. Cell-autonomous enhancement of lipolysis by cooling temperatures.

A. The ratio of cellular triacylglycerol to diacylglycerol (DAG) in primary inguinal adipocytes treated at the indicated temperatures (37 °C, 31 °C, and 27 °C) in the presence of vehicle or norepinephrine (NE) at 0.1 μM. n = 6. Data are presented as mean ± s.e.m. Two-way ANOVA with Šídák's multiple comparisons test.

B. Representative bright-field images of differentiated primary brown adipocytes treated with increasing concentrations of norepinephrine (NE) at 0.1, 0.5, and 1.0 μM at 37 °C or 27 °C for 10 hours. Scale bar: 210 μm.

C. Glycerol release from differentiated primary brown adipocytes treated with increasing concentrations of NE (0.1, 0.5, and 1.0 μM) at 37 °C or 27 °C. Lipolysis was quantified as glycerol release normalized to protein amount. n = 3. Data are presented as mean ± s.e.m. Unpaired t-test.

D. Representative BODIPY staining of differentiated brown adipocytes treated at the indicated temperatures (37 °C, 31 °C, and 27 °C) with vehicle or norepinephrine (NE) at 0.1 μM. Scale bar: 130 μm.

bioRxiv preprint doi: https://doi.org/10.64898/2026.10.22.760470; this version posted October 27, 2026. The copyright holder for this preprint (which was not certified by peer review) is the author/funder, who has granted bioRxiv a license to display the preprint in perpetuity. It is made available under a CC-BY 4.0 International license.

E. Glycerol release from differentiated brown adipocytes treated at the indicated temperatures (37 °C, 31 °C, and 27 °C) with vehicle or norepinephrine (NE) at 0.1 µM. Lipolysis was quantified as glycerol release normalized to total protein. n = 3. Data are presented as mean ± s.e.m. One-way ANOVA followed by Tukey's multiple comparisons test.

F. Heatmap showing changes in medium BSA-bound free fatty acids (FFA), cellular acyl-carnitines and cellular indicated metabolites following NE treatment for 10 hours at 27ºC or 37ºC. Values are presented as Z-scores. n = 6 per group. Unpaired t-test.

G. Glycerol release from differentiated brown adipocytes treated at 37 °C or 27 °C with vehicle (DMSO) or Atglistatin (20 µM), in the presence or absence of norepinephrine (NE) at 0.1 µM for 10 h. n =6 per group. Data are normalized to total protein and presented as mean ± s.e.m. Two-way ANOVA with Šídák's multiple comparisons test.

H. Free fatty acids (FFA) release from immortalized brown adipocytes treated at 37 °C or 27 °C with vehicle (DMSO) or Atglistatin (20 µM), in the presence or absence of norepinephrine (NE, 0.1 µM) for 10 h. n =6 per group. Data are normalized to total protein and presented as mean ± s.e.m. Two-way ANOVA with Šídák's multiple comparisons test.

bioRxiv preprint doi: https://doi.org/10.64898/2026.10.22.760470; this version posted October 27, 2026. The copyright holder for this preprint (which was not certified by peer review) is the author/funder, who has granted bioRxiv a license to display the preprint in perpetuity. It is made available under a CC-BY 4.0 International license.

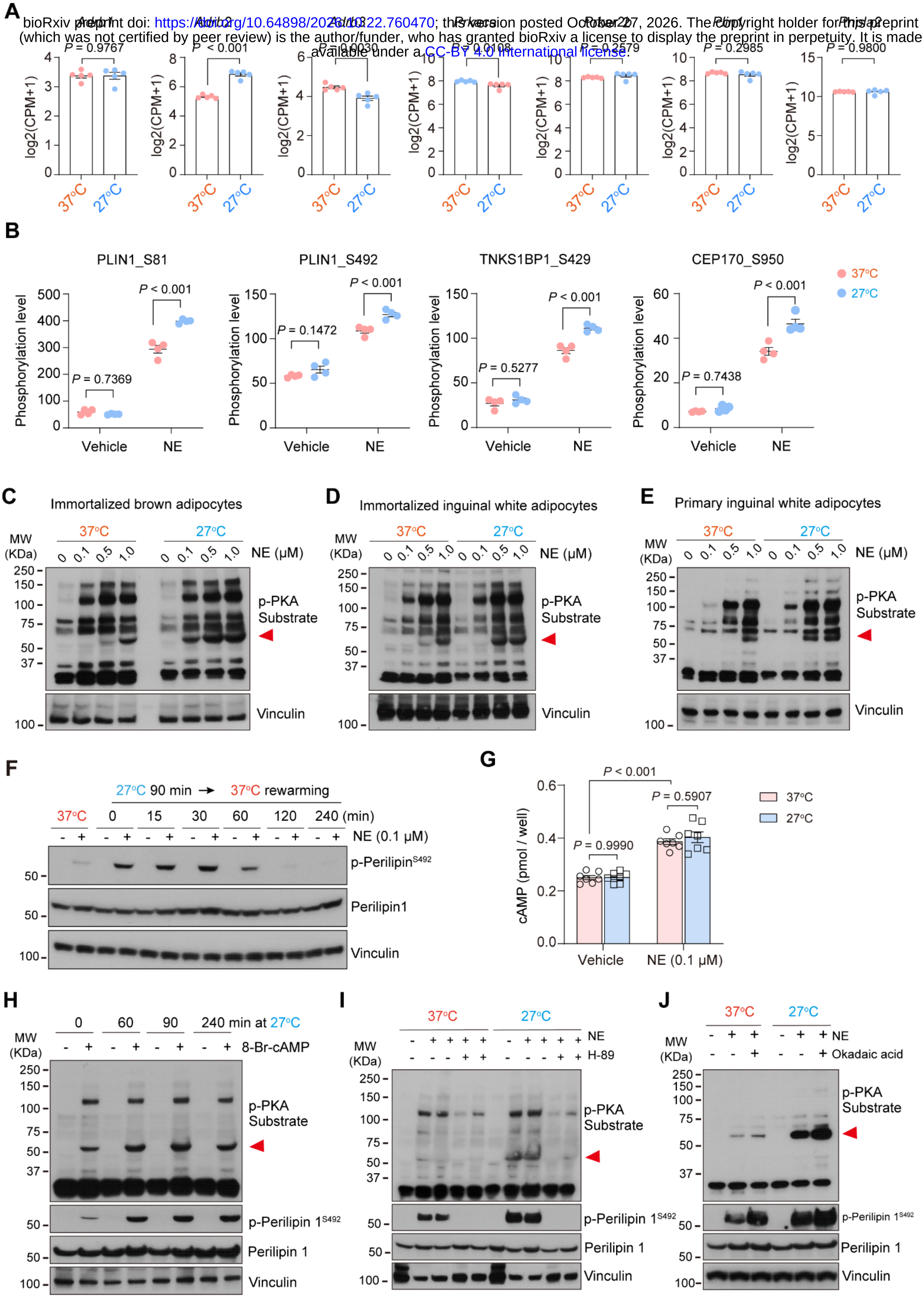

Supplementary Figure 3. Cooling temperatures selectively amplify PKA-perilipin 1 signaling.

bioRxiv preprint doi: https://doi.org/10.64898/2026.10.22.760470; this version posted October 27, 2026. The copyright holder for this preprint (which was not certified by peer review) is the author/funder, who has granted bioRxiv a license to display the preprint in perpetuity. It is made available under aCC-BY 4.0 International license.

A. Immortalized brown adipocytes were pre-incubated at 27 °C for 1 h prior to stimulation with norepinephrine (NE) at 0.1 μM for 3 h. Cells were harvested for RNA sequencing (Plasmidsaurus). Expression levels of genes related to lipolysis are presented as log2-transformed CPM values log2(CPM + 1). $n = 5$. Data are shown as mean ± s.e.m.. Statistical significance was determined using an unpaired two-tailed Student's t-test on log-transformed CPM values.

B. Quantification of phosphorylation levels of representative sites identified from phosphoproteomic analysis, including perilipin 1 (S81 and S492), TNKS1BP1 (S429), and CEP170 (S950), under vehicle or norepinephrine (NE) at 0.1 μM stimulation at 37 °C or 27 °C. $n = 4$ per group. Data are presented as mean ± s.e.m. Two-way ANOVA with Šídák's multiple comparisons test.

C. Differentiated brown adipocytes were pre-incubated at 37 °C or 27 °C for 90 min, followed by stimulation with norepinephrine (NE) at 0.1, 0.5, and 1.0 μM for 10 min. Immunoblot analysis shows phosphorylation of the indicated PKA substrates. Vinculin was used as a loading control.

D. Immortalized inguinal WAT-derived differentiated adipocytes were treated as in (**C**). Immunoblot analysis shows phosphorylation of PKA substrates. Vinculin was used as a loading control.

E. Primary inguinal WAT-derived adipocytes were treated as in (**C**). Immunoblot analysis shows phosphorylation of PKA substrates. Vinculin was used as a loading control.

F. Reversibility of cooling-induced perilipin 1 phosphorylation after rewarming. Immunoblot analysis of perilipin 1^{S492} phosphorylation in adipocytes following 90 min cooling at 27°C and subsequent rewarming at 37°C for up to 240 min. Vinculin was used as a loading control.

G. Intracellular cAMP levels in adipocytes incubated at 27°C or 37°C for 90 min. Cells were treated with norepinephrine (NE; 0.1 μM) for 10 min before harvest. No IBMX is used to inhibit phosphodiesterase activity. $n = 7$ per group. Data are presented as mean ± s.e.m. Two-way ANOVA with Šídák's multiple comparisons test.

H. Differentiated brown adipocytes were pre-incubated at 27 °C for 0 min, 60 min, 90 min or 240 min, followed by stimulation with 8-Br-cAMP (200 μM) for 10 min. Immunoblot analysis shows phosphorylation of PKA substrates, phospho-perilipin $1^{S492,}$ and total perilipin 1. Vinculin was used as a loading control.

I. Differentiated brown adipocytes were pre-incubated at 37 °C or 27 °C for 90 min and then treated with the PKA inhibitor H-89 (15 μM) or vehicle for 20 min before stimulation with norepinephrine (NE; 0.1 μM) or vehicle. Immunoblot analysis shows phosphorylation of PKA substrates, phospho- perilipin $1^{S492,}$ and total perilipin 1. Vinculin was used as a loading control.

J. Differentiated brown adipocytes were pre-incubated at 37 °C or 27 °C for 60 min, followed by treatment with okadaic acid (50 nM) for 30 min. Cells were then stimulated with norepinephrine (NE) at 0.1 μM for 10 min prior to harvest. Immunoblot analysis shows phosphorylation of PKA substrates, phospho-perilipin $1^{S492,}$ and total perilipin 1. Vinculin was used as a loading control.

bioRxiv preprint doi: https://doi.org/10.64898/2026.10.22.760470; this version posted October 27, 2026. The copyright holder for this preprint (which was not certified by peer review) is the author/funder, who has granted bioRxiv a license to display the preprint in perpetuity. It is made available under a CC-BY 4.0 International license.

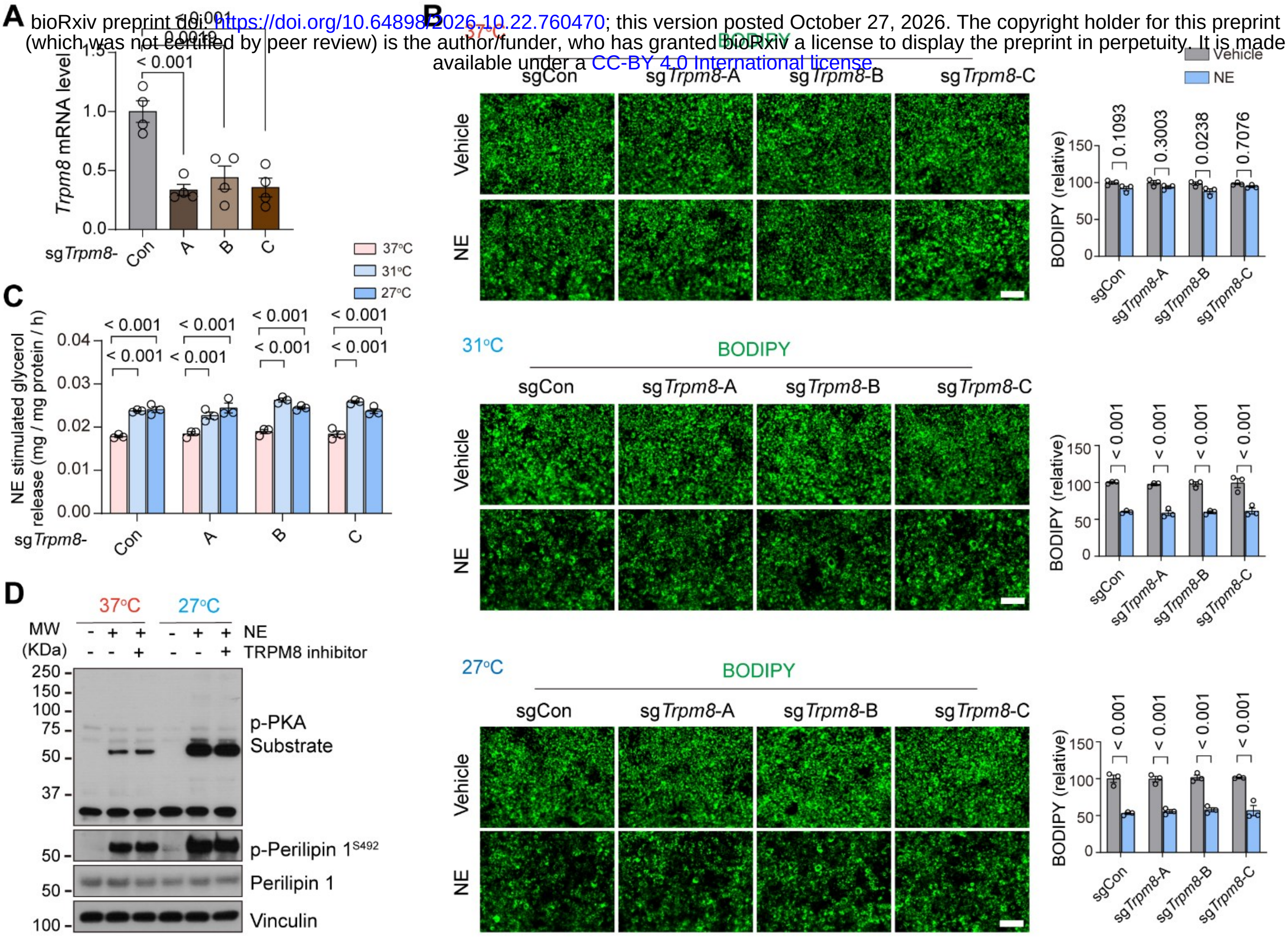

Supplementary Figure 4. Cooling temperatures selectively augment PKA signaling independent of the TRP channels.

A. mRNA levels of *Trpm8* in adipocytes expressing control sgRNA (sgCon) or three independent sgRNAs targeting *Trpm8* (sg*Trpm8*-A, -B, and -C). $n = 4$ per group. Data are presented as mean ± s.e.m. One-way ANOVA followed by Tukey's multiple comparisons test.

B. Representative BODIPY staining of adipocytes expressing sgCon or sg*Trpm8* constructs under vehicle or norepinephrine (NE) at 0.1 µM for 10 hours at the indicated temperatures. Scale bar: 130 µm. Quantification of BODIPY fluorescence intensity is shown on the right. $n = 3$ per group. Data are presented as mean ± s.e.m. Two-way ANOVA with Šídák's multiple comparisons test.

C. NE-induced glycerol release in adipocytes expressing sgCon or sg*Trpm8* constructs treated at 37 °C, 31 °C, or 27 °C after NE treatment at 0.1 µM for 10 hours. $n = 3$ per group. Data are presented as mean ± s.e.m. Two-way ANOVA with Šídák's multiple comparisons test.

D. Differentiated brown adipocytes were pre-treated with the TRPM8 inhibitor BCTC (10 µM) or vehicle (DMSO) for 10 min, followed by incubation at 37 °C or 27 °C for 90 min and subsequent stimulation by norepinephrine (NE) at 0.1 µM for 10 min. Immunoblot analysis shows phosphorylation of PKA substrates, phospho- perilipin 1^{S492,} and total perilipin 1. Vinculin was used as a loading control.

bioRxiv preprint doi: https://doi.org/10.64898/2026.10.22.760470; this version posted October 27, 2026. The copyright holder for this preprint (which was not certified by peer review) is the author/funder, who has granted bioRxiv a license to display the preprint in perpetuity. It is made available under a CC-BY 4.0 International license.

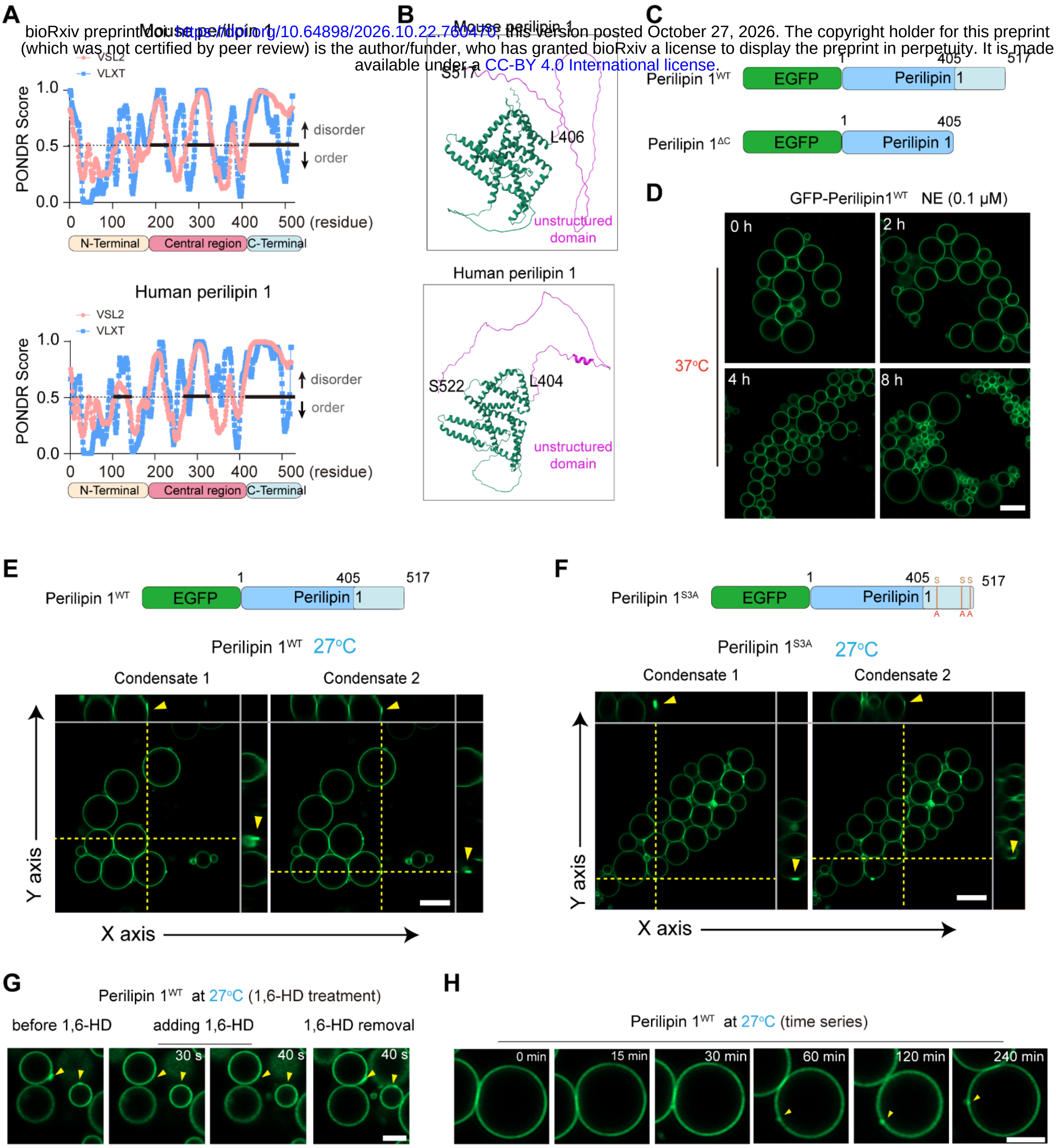

Supplementary Figure 5. Cooling temperatures induce the formation of perilipin 1 condensates.

bioRxiv preprint doi: https://doi.org/10.64898/2026.10.22.760470; this version posted October 27, 2026. The copyright holder for this preprint (which was not certified by peer review) is the author/funder, who has granted bioRxiv a license to display the preprint in perpetuity. It is made available under a CC-BY 4.0 International license.

A. Prediction of intrinsically disordered regions in mouse (Top) and human (Bottom) perilipin 1 using the PONDR algorithm (pink: VSL2, blue: VLXT) with bold lines indicating predicted disordered regions. The bottom schematic illustrates domain annotations of the human perilipin 1 protein (N-terminal, Central region, and C-terminal).

B. Structural prediction of the mouse (Top) and human (Bottom) perilipin 1 protein by Alphafold3, highlighting a folded N-terminal domain and an extended C-terminal unstructured region. Residues after L406 (mouse) or L404 (human) form a predicted intrinsically disordered region (IDR), lacking defined secondary structure.

C. Schematic of GFP-tagged perilipin 1 constructs, including full-length perilipin 1 (Perilipin 1^{WT}), C-terminal truncation (Perilipin $1^{\Delta C}$).

D. Representative GFP-perilipin 1 images in differentiated adipocytes treated with norepinephrine (NE; 0.1 µM) at 37°C for the indicated times, up to 8 h. Scale bar: 4 µm.

E. Top: Schematic of GFP-tagged full-length perilipin 1 (Perilipin 1^{WT}). Bottom: Representative orthogonal visualizations of indicated condensates at 27°C, reconstructed from confocal Z-stack images. Yellow arrows indicate perilipin1 condensates. Scale bar: 4 µm.

F. Top: Schematic of GFP-tagged C-terminal PKA phosphorylation-deficient mutant (Perilipin 1^{S3A}; S433A/S492A/S517A). Bottom: Representative orthogonal visualizations of indicated condensates at 27°C, reconstructed from confocal Z-stack images. Yellow arrows indicate perilipin1 condensates. Scale bar: 4 µm.

G. Cooling-induced perilipin 1 puncta formation in response to 1,6-hexanediol (1,6-HD, 5%) treatment. Representative images showing GFP-perilipin 1 puncta in adipocytes treated at 27 °C before, after 1,6-HD treatment for 30 or 40 seconds, and after the withdrawal of 1,6-HD. Yellow arrows indicate perilipin1 condensates. Scale bar: 2 µm.

H. Time-lapse live-cell imaging of GFP-perilipin 1 condensate formation at 27˚C. Time points are shown on the right. Yellow arrows indicate perilipin1 condensates. Scale bar: 2 µm.

bioRxiv preprint doi: https://doi.org/10.64898/2026.10.22.760470; this version posted October 27, 2026. The copyright holder for this preprint (which was not certified by peer review) is the author/funder, who has granted bioRxiv a license to display the preprint in perpetuity. It is made available under a CC-BY 4.0 International license.

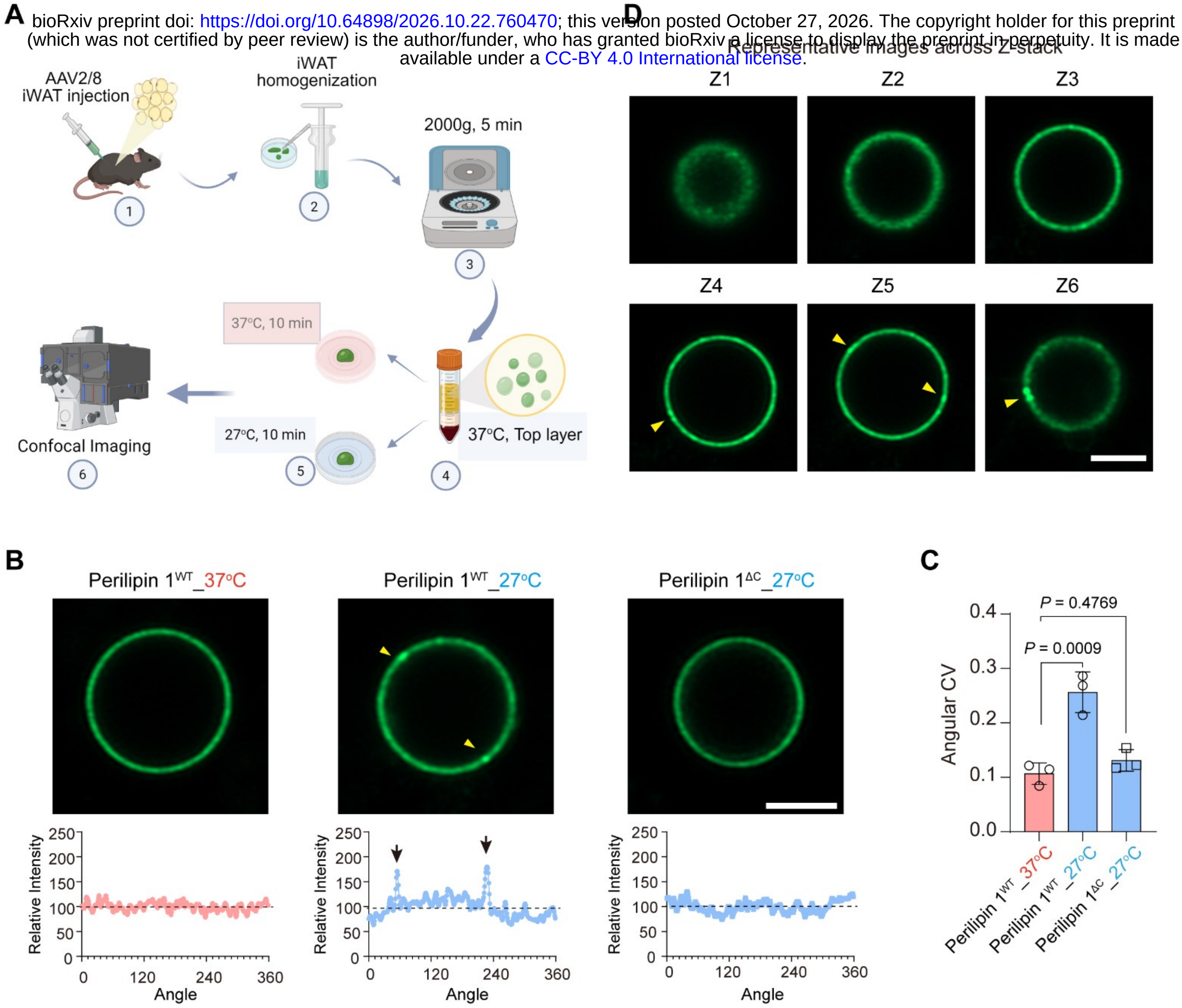

Supplementary Figure 6. Cooling induces the C-terminal-dependent perilipin 1 condensate formation on lipid droplets.

A. Workflow for assessing perilipin 1 condensation on lipid droplets. iWAT of 8-week-old male mice was subcutaneously injected with AAV2/8 expressing GFP-PLIN1. Three weeks after injection, lipid droplets were isolated from iWAT, placed in glass-bottom dishes, and incubated at 37 °C or 27 °C for 10 min, followed immediately by confocal imaging.

B. Top: Representative GFP images of full-length GFP-PLIN1 (Perilipin 1^{WT}) on isolated lipid droplets from cultured adipocytes incubated at 37 °C (left) or 27 °C (middle), and GFP–perilipin $1^{\Delta C}$ on isolated lipid droplets incubated at 27 °C (right). Bottom: Corresponding angular distributions of GFP fluorescence intensity around the lipid droplets, using Fiji software. Arrowheads indicate perilipin 1 condensates on lipid droplets.

C. Coefficient of variation (CV) of perilipin 1 fluorescence intensity on lipid droplet surface under the indicated conditions. *n*= 3 independent experiment per group. One-way ANOVA followed by Tukey's multiple comparisons test.

D. Representative Z-stack images of perilipin 1 condensates from (**B**). Scale bar: 2 µm. Arrowheads indicate perilipin 1 condensates on lipid droplets.

bioRxiv preprint doi: https://doi.org/10.64898/2026.10.22.760470; this version posted October 27, 2026. The copyright holder for this preprint (which was not certified by peer review) is the author/funder, who has granted bioRxiv a license to display the preprint in perpetuity. It is made available under a CC-BY 4.0 International license.

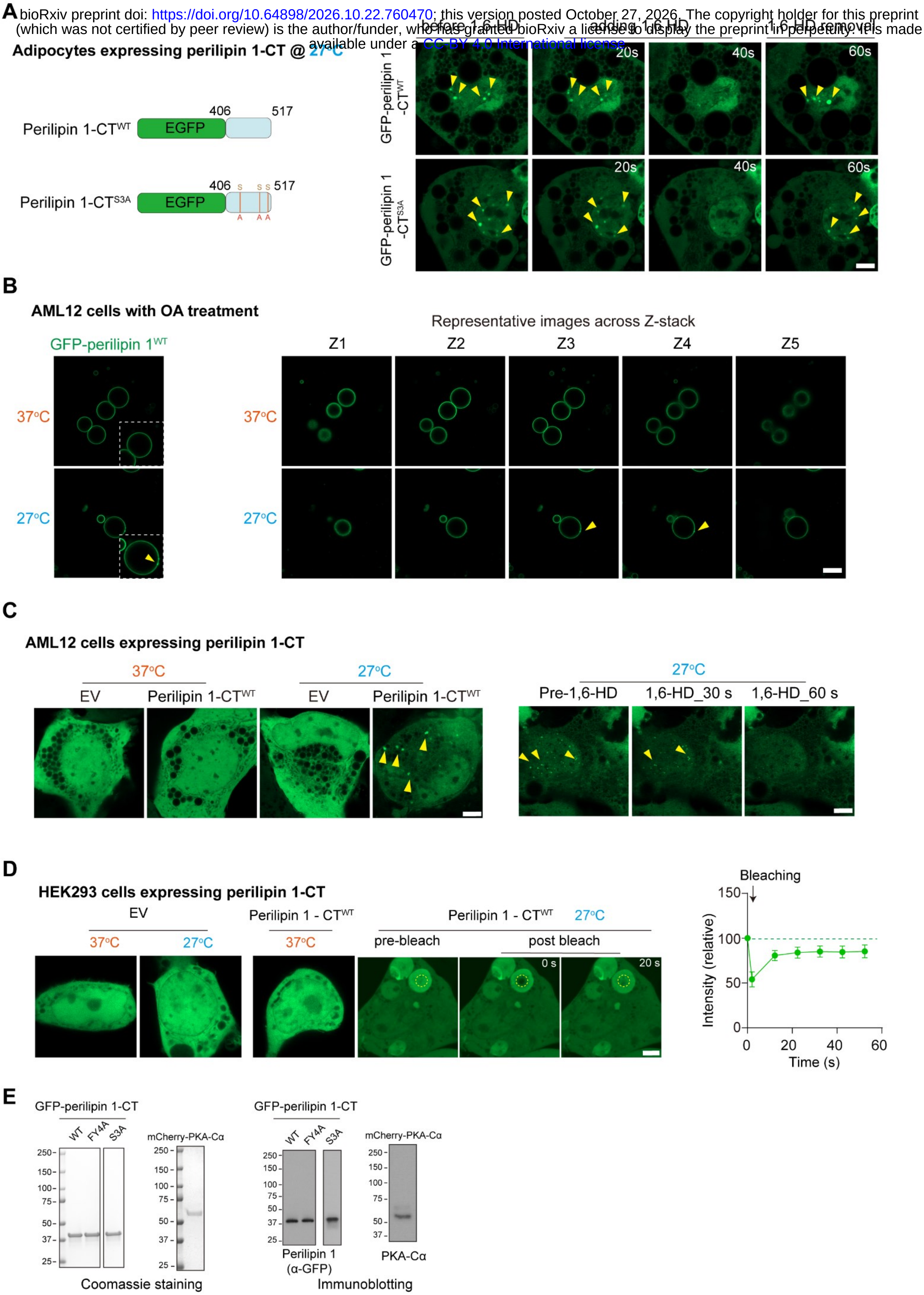

Supplementary Figure 7. The C-terminal domain of perilipin 1 is sufficient for cooling-induced LLPS.

bioRxiv preprint doi: https://doi.org/10.64898/2026.10.22.760470; this version posted October 27, 2026. The copyright holder for this preprint (which was not certified by peer review) is the author/funder, who has granted bioRxiv a license to display the preprint in perpetuity. It is made available under a CC-BY 4.0 International license.

A. Left: Schematic of GFP-tagged constructs containing the C-terminal region of perilipin 1. Right: Representative images of adipocytes expressing GFP-perilipin 1-CT^{WT} (Top) or GFP-perilipin 1-CT^{S3A} (S433/492/517A; Bottom) at 27 °C for 8 hours before, during, and after treatment with 1,6-hexanediol (1,6-HD, 5%). Perilipin 1 condensates (arrowheads) rapidly dissolved upon 1,6-HD treatment and reformed after removal. Scale bar: 4 µm.

B. Left: Representative GFP–perilipin 1 images of AML12 hepatocytes expressing GFP–perilipin 1. Cells were treated with oleic acid (OA) for 48 h to induce lipid-droplet formation and subsequently incubated at 37°C or 27°C. Arrowheads indicate perilipin 1 condensates. Right: Representative serial Z-stack images of corresponding GFP–perilipin 1 hepatocytes. Scale bar: 4 µm.

C. Left: Representative images of AML12 cells expressing GFP-perilipin 1-CT^{WT} or GFP control after incubation at 37 °C or 27 °C for 8 hours. Right: Representative images of AML12 cells expressing GFP-perilipin 1-CT^{WT} cultured at 27 °C before and after treatment with 1,6-hexanediol (1,6-HD, 5%). Scale bar: 4 µm. Arrowheads indicate perilipin 1 condensates.

D. Left: HEK293 cells expressing GFP–perilipin 1-CT or GFP control were cultured at 27 °C for 8 h or 37 °C as a control. FRAP analysis of perilipin 1-CT condensates at 27 °C shows rapid fluorescence recovery. Scale bar: 4 µm. Right: Quantification of the bleaching area is shown on the right. $n = 3$.

E. Left: Coomassie Brilliant Blue staining of purified GFP–perilipin 1-CT^{WT}, GFP–perilipin 1-CT^{FY4A}, GFP–perilipin 1-CT^{S3A}, and mCherry-PKA-Cα. Right: Immunoblotting with anti-GFP or PKA- Cα confirmed protein identity.

bioRxiv preprint doi: https://doi.org/10.64898/2026.10.22.760470; this version posted October 27, 2026. The copyright holder for this preprint (which was not certified by peer review) is the author/funder, who has granted bioRxiv a license to display the preprint in perpetuity. It is made available under a CC-BY 4.0 International license.

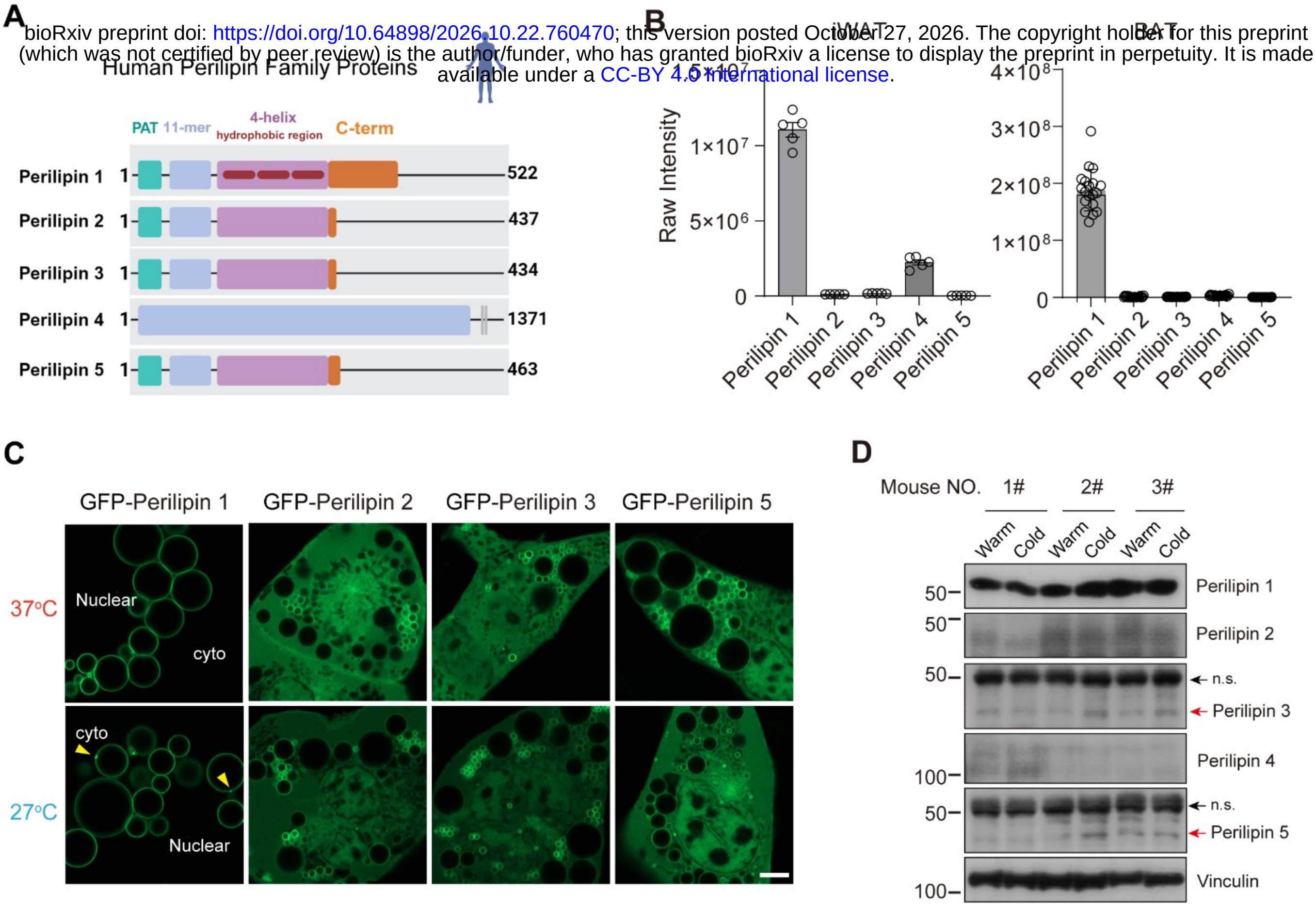

Supplementary Figure 8. The distinct feature of perilipin 1 among perilipin family proteins.

A. Schematic structural comparison of human perilipin family members (Perilipin 1, 2, 3, 4, and 5), highlighting the conserved domains, including the PAT domain, 11-mer repeats, and amphipathic helices. Note that perilipin 1 is distinct from other family members in the C-terminal extension and three hydrophobic regions. PLIN4 exhibits an extended central repeat region. Protein lengths are indicated on the right.

B. Left: Protein abundance (raw intensity) of perilipin family members in iWAT from a publicly available dataset. Right: Protein abundance (raw intensity) of perilipin family members in BAT from a publicly available dataset.

C. Representative images of adipocytes expressing GFP-tagged perilipin 1, perilipin 2, perilipin 3, or perilipin 5 following incubation at 37°C or 27°C. Arrowheads indicate perilipin 1 condensates on lipid droplets. Scale bar: 4 µm.

D. Immunoblot analysis showing no noticeable changes in perilipin 1–5 protein expression in iWAT from the ice-pad-treated side (cold) compared with warm-pad-treated side (warm). n.s., non-specific band.

bioRxiv preprint doi: https://doi.org/10.64898/2026.10.22.760470; this version posted October 27, 2026. The copyright holder for this preprint (which was not certified by peer review) is the author/funder, who has granted bioRxiv a license to display the preprint in perpetuity. It is made available under a CC-BY 4.0 International license.

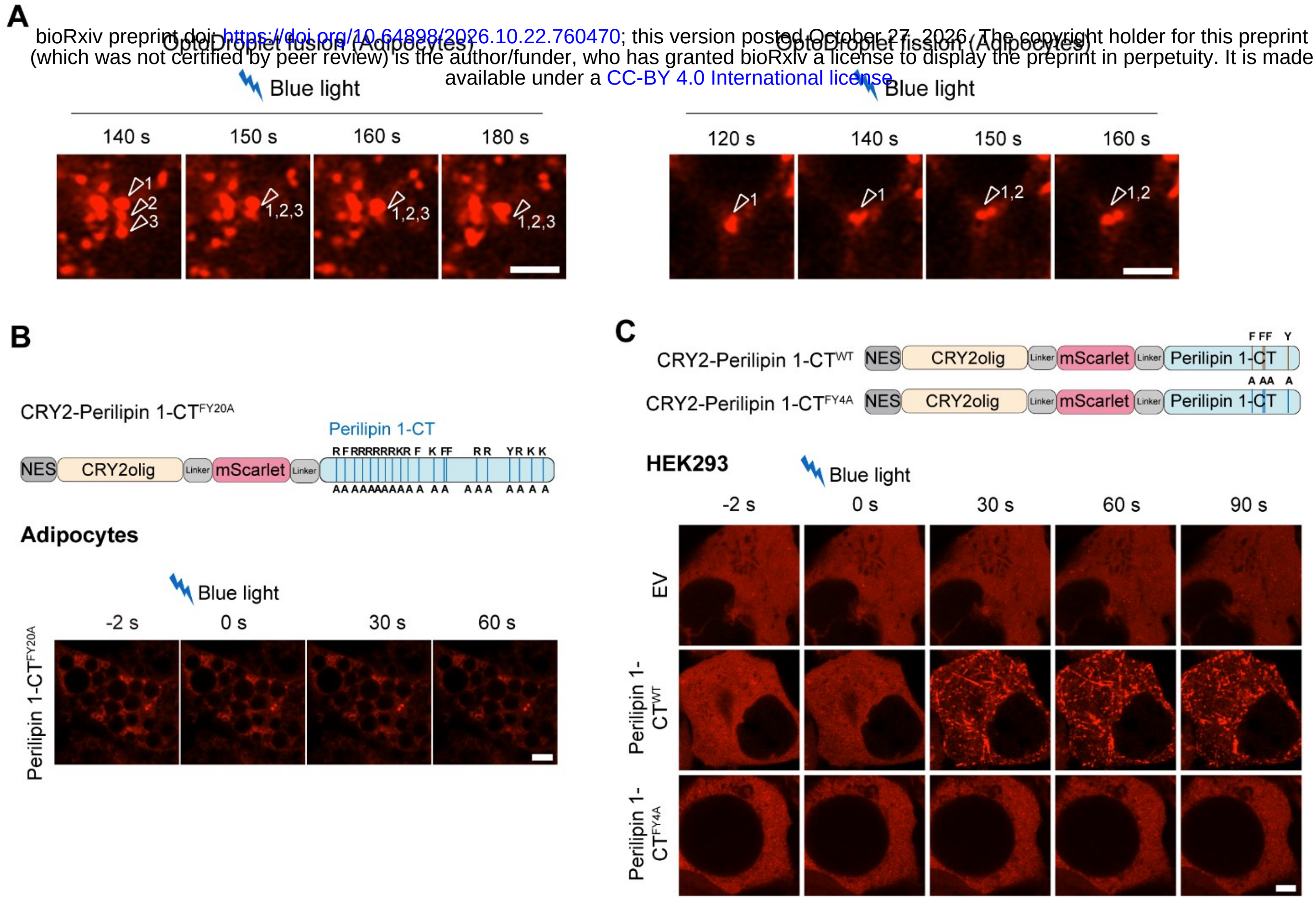

Supplementary Figure 9. The structural requirement for light-induced perilipin 1 phase separation.

A. Time-lapse imaging of optogenetic perilipin 1-CT droplets in adipocytes following blue light stimulation (see Figure 4D). Left: Representative images showing fusion of multiple condensates over time from 140 to 180 sec. Right: Representative images showing fission events from 120 to 160 s, where individual condensates split into smaller droplets. Arrowheads indicate tracked condensates. Scale bar: 2 µm.

B. Top: Schematic of the CRY2-based optogenetic constructs used to test the clustering of a phase separation-deficient perilipin 1 mutant. The mutant (perilipin 1-CT^{FY20A}), in which five aromatic residues (F418, F479, F495, F496, Y510) and fifteen lysine/arginine residues (R408, R419, R436, R445, R450, R454, R457, K470, R476, K486, R497, R507, R514, K515, K516) were substituted with alanine, was fused to CRY2olig–mScarlet with a nuclear export signal. Bottom: Representative images in differentiated brown adipocytes expressing perilipin 1-CT^{FY20A} before (−2 s) and after blue light stimulation at 0, 30, and 60 s. Scale bar: 4 µm.

C. Top: Schematic of the CRY2-based optogenetic constructs used in HEK293 cells. Bottom: Representative images in HEK293 cells expressing empty vector (EV), perilipin 1-CT^{WT}, or perilipin 1-CT^{FY4A} before (−2 s) and after blue light stimulation at 0, 30, 60, and 90 s. Scale bar: 4 µm.

bioRxiv preprint doi: https://doi.org/10.64898/2026.10.22.760470; this version posted October 27, 2026. The copyright holder for this preprint (which was not certified by peer review) is the author/funder, who has granted bioRxiv a license to display the preprint in perpetuity. It is made available under a CC-BY 4.0 International license.

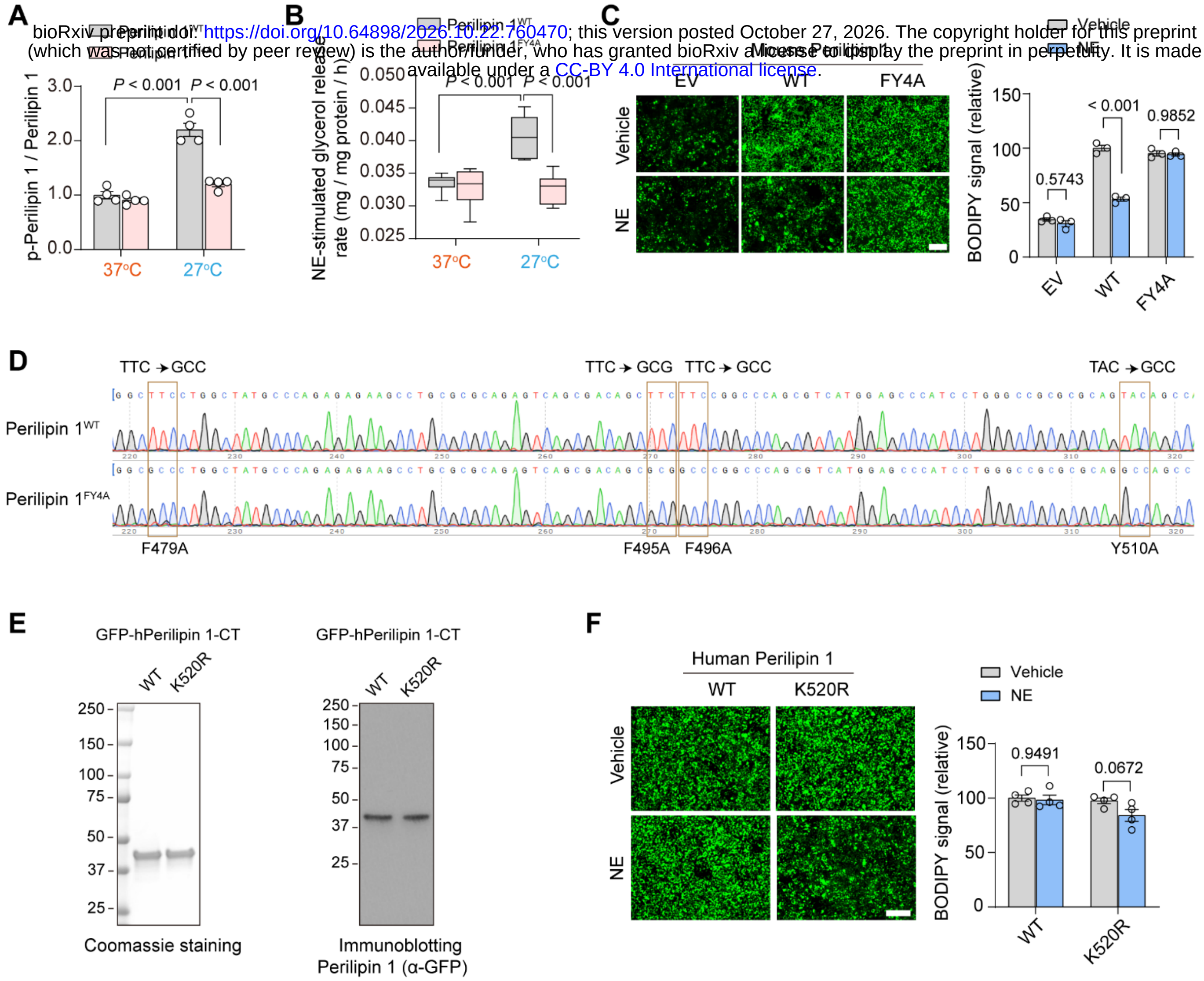

Supplementary Figure 10. Perilipin 1 LLPS is required for cooling-induced lipolysis.

A. Quantification of phospho-perilipin 1 levels shown in Fig. 4E. $n = 4$ per group. Data are presented as mean ± s.e.m. Two-way ANOVA with Šídák's multiple comparisons test.

B. Lipolysis was assessed by measuring glycerol release into the conditioned media after 8 hours of norepinephrine (NE, 0.1 µM) stimulation. Glycerol levels were normalized to total protein. $n = 6$ per group. Data are presented as mean ± s.e.m. Two-way ANOVA with Šídák's multiple comparisons test.

C. Left: Representative BODIPY staining of *Plin1* KO adipocytes reconstituted with an empty vector (EV), mouse perilipin 1^{WT}, or the phase separation–deficient mutant perilipin 1^{FY4A} treated with vehicle or norepinephrine (NE, 0.1 µM) at 27 °C. Scale bar: 130 µm. Right: Quantification of BODIPY fluorescence intensity is shown on the right. $n = 3$ per group. Data are presented as mean ± s.e.m. Two-way ANOVA followed by with Šídák's multiple comparisons test.

D. Sanger sequencing confirmation of the perilipin 1^{FY4A} knock-in mouse allele, carrying the F479A, F495A, F496A, and Y510A substitutions.

bioRxiv preprint doi: https://doi.org/10.64898/2026.10.22.760470; this version posted October 27, 2026. The copyright holder for this preprint (which was not certified by peer review) is the author/funder, who has granted bioRxiv a license to display the preprint in perpetuity. It is made available under a CC-BY 4.0 International license.

E. Left: Coomassie Brilliant Blue staining of purified GFP-tagged human perilipin 1 C-terminal proteins (GFP-perilipin 1-CT^{WT} and GFP-perilipin 1-CT^{K520R}). Right: Immunoblotting with anti-GFP confirmed their protein identity.

F. Left: Representative BODIPY staining of *Plin1* KO adipocytes reconstituted with human perilipin 1^{WT} or perilipin 1^{K520R} treated with vehicle or norepinephrine (NE) at 37 °C. Scale bar: 130 µm. Right: Quantification of BODIPY fluorescence intensity. $n = 4$ per group. Data are presented as mean ± s.e.m. Two-way ANOVA followed by Šídák's multiple comparisons test.

bioRxiv preprint doi: https://doi.org/10.64898/2026.10.22.760470; this version posted October 27, 2026. The copyright holder for this preprint (which was not certified by peer review) is the author/funder, who has granted bioRxiv a license to display the preprint in perpetuity. It is made available under a CC-BY 4.0 International license.

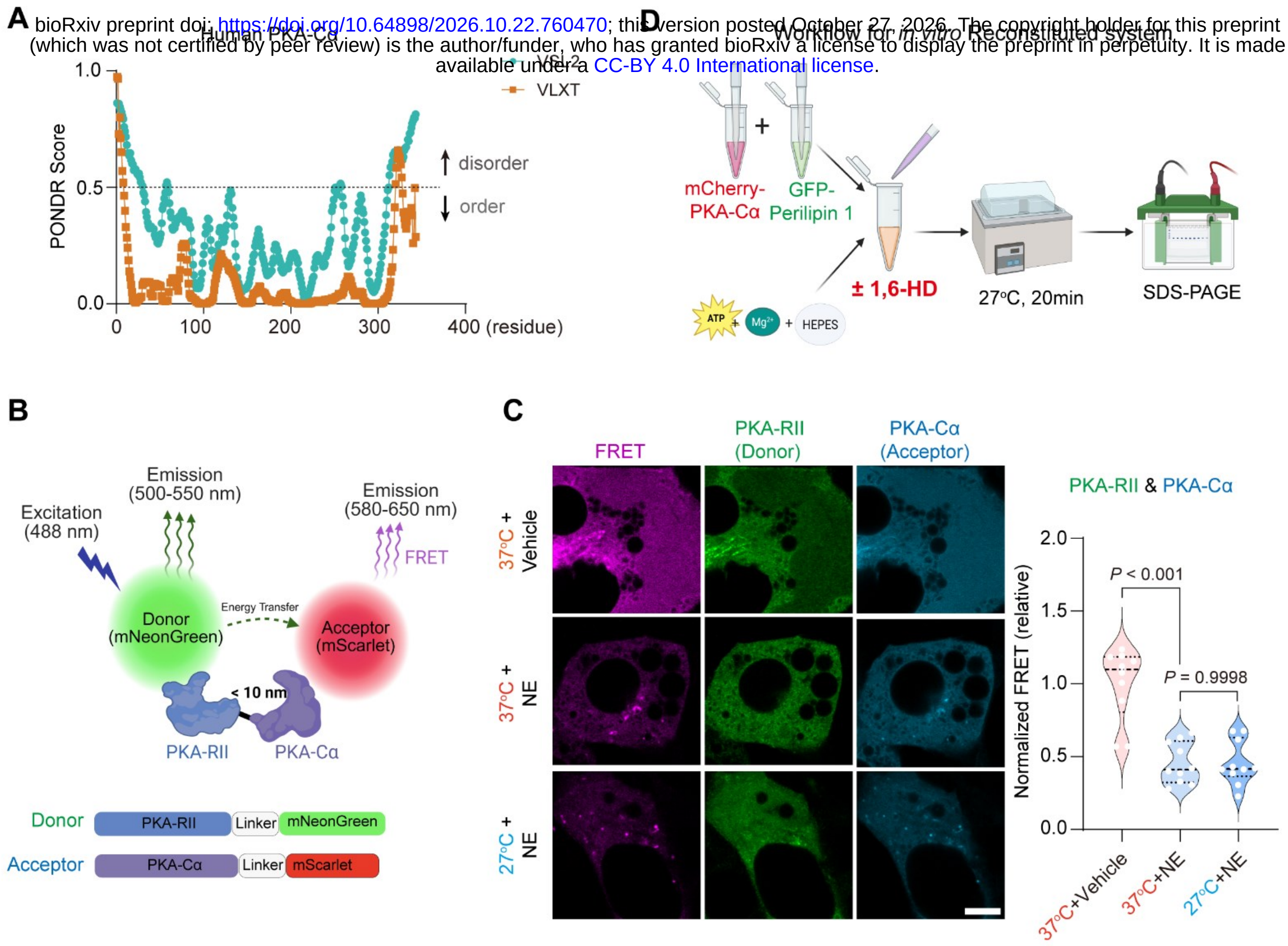

Supplementary Figure 11. Cooling temperature did not alter the proximity of PKA-Cα and PKA-RII in adipocytes.

A. Prediction of intrinsically disordered regions in human PKA-Cα using the PONDR algorithm (pink: VSL2, blue: VLXT).

B. Schematic illustration of the Förster resonance energy transfer (FRET) system using PKA-RII-mNeonGreen as the donor and PKA-Cα–mScarlet as the acceptor. Upon excitation at 488 nm, energy transfer occurs when the two proteins are within <10 nm, leading to acceptor emission (580–650 nm). Donor emission (500–550 nm) and acceptor signals were used to quantify FRET efficiency.

C. Left: Representative images of FRET-based proximity assay between PKA-RII and PKA-Cα in adipocytes. Differentiated brown adipocytes stably expressing PKA-RII-mNeonGreen (Donor) and PKA-Cα–mScarlet (Acceptor) were incubated at 37 °C or 27 °C for 90 min, followed by NE stimulation at 0.1 µM for 1 min. Scale bar: 5 µm. Right: Quantification of FRET signals. n = 10 adipocytes per group. Data are presented as mean ± s.e.m. One-way ANOVA followed by Tukey's multiple comparisons test.

D. Workflow of the *in vitro* reconstituted system to assess perilipin 1 phosphorylation by PKA-Cα. Recombinant GFP–perilipin 1 and mCherry–PKA-Cα proteins were mixed in a buffer containing HEPES, Mg^{2+}, and ATP, in the presence or absence of 1,6-HD at 5%. Samples were incubated at 27 °C for 20 min prior to SDS–PAGE analysis to assess perilipin 1 phosphorylation under condensate-forming conditions.

bioRxiv preprint doi: https://doi.org/10.64898/2026.10.22.760470; this version posted October 27, 2026. The copyright holder for this preprint (which was not certified by peer review) is the author/funder, who has granted bioRxiv a license to display the preprint in perpetuity. It is made available under a CC-BY 4.0 International license.

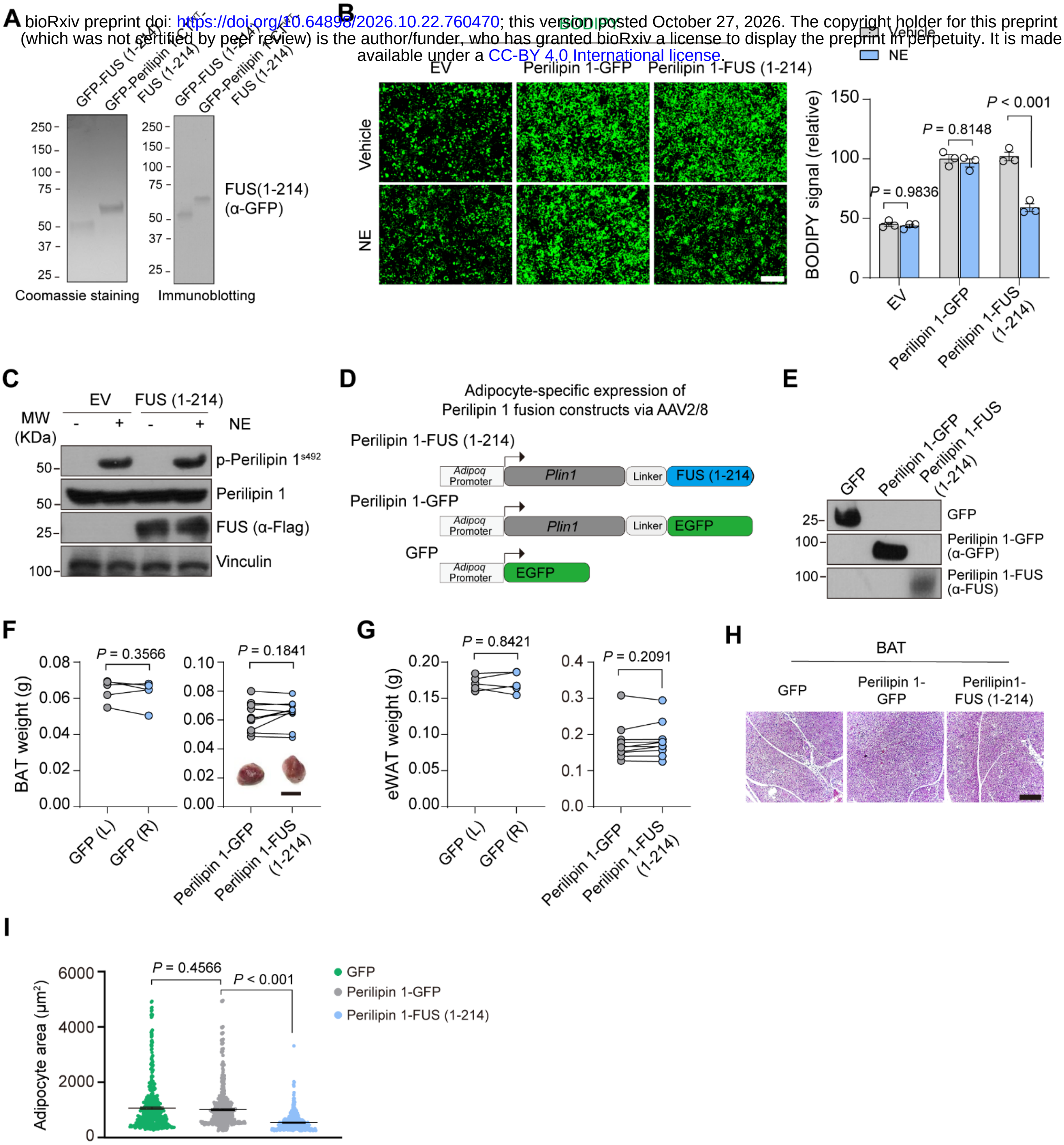

Supplementary Figure 12. Enhanced perilipin 1 phase separation promotes lipolysis.

bioRxiv preprint doi: https://doi.org/10.64898/2026.10.22.760470; this version posted October 27, 2026. The copyright holder for this preprint (which was not certified by peer review) is the author/funder, who has granted bioRxiv a license to display the preprint in perpetuity. It is made available under a CC-BY 4.0 International license.

A. Left: Coomassie Brilliant Blue staining of purified GFP–FUS (1–214) and GFP–perilipin 1-CT–FUS (1–214). Right: Immunoblotting with anti-GFP confirmed the protein identity.

B. Left: Representative BODIPY staining of perilipin 1-knockout adipocytes reconstituted with an empty vector (EV), perilipin 1–GFP, or perilipin 1–FUS (1–214) in response to the treatment with vehicle or norepinephrine (NE) at 0.1 µM for 8 hours. Scale bar: 130 µm. Right: Quantification of BODIPY fluorescence intensity. n = 3 per group. Data are presented as mean ± s.e.m. Two-way ANOVA with Šídák's multiple comparisons test.

C. Differentiated adipocytes expressing empty vector (EV) or Flag–FUS (1–214) were stimulated with norepinephrine (NE) at 0.1 µM for 10 min. Immunoblot analysis shows phospho- perilipin 1^{S492}, total perilipin 1, and FUS. Vinculin was used as a loading control.

D. Schematic of AAV2/8 vectors driven by the adiponectin (*Adipoq*) promoter to express perilipin 1–FUS (1–214), perilipin 1–GFP, or GFP that were used *in vivo*.

E. Immunoblot analysis of GFP, perilipin 1-GFP, and perilipin 1–FUS (1–214) in adipose tissue.

F. Brown adipose tissue weight from matched depots of mice expressing GFP, perilipin 1-GFP, or perilipin 1-FUS (1–214) following bilateral subcutaneous injection. Each data point represents an individual depot, with the left or right depots from the same mouse. Representative BAT images are shown at the bottom inset. n = 5 mice for control group, n = 10 mice for FUS group. Paired t-test.

G. Epididymal white adipose tissue (eWAT) weight from matched depots of mice expressing GFP, perilipin 1-GFP, or perilipin 1-FUS (1–214) following bilateral subcutaneous injection. Each data point represents an individual depot, with the left or right depots from the same mouse. n = 5 mice for control group, n = 10 mice for FUS group. Paired t-test.

H. Representative hematoxylin and eosin (H&E) staining of BAT sections from matched depots of mice expressing GFP, perilipin 1-GFP, or perilipin 1-FUS (1–214) following bilateral subcutaneous injection. Scale bar: 210 µm.

I. Quantification of adipocyte area shown in Fig. 6K. n =400 cells per group from 3 mice. One-way ANOVA followed by Tukey's multiple comparisons test.

Table S1.

bioRxiv preprint doi: https://doi.org/10.64898/2026.10.22.760470; this version posted October 27, 2026. The copyright holder for this preprint (which was not certified by peer review) is the author/funder, who has granted bioRxiv a license to display the preprint in perpetuity. It is made available under a CC-BY 4.0 International license.

Temperature-dependent regulation of norepinephrine-induced phosphopeptides

	Phosphorylation level	37 ºC + Vehicle	27 ºC + Vehicle	37 ºC + NE	27 ºC + NE
Up in 27 ºC	Perilipin 1 S81	59.6125	51.4825	294.12	398.495
	Perilipin 1 S492	58.175	65.4125	108.735	127.0975
	CEP170 S950	7.1475	8.5275	34.12	46.4775
	TNKS1BP1 S429	27.0525	30.9775	86.265	111.175
Down in 27 ºC	STMN1 S63	68.36	69.305	250.15	129.055
	STMN1 S16	24.3475	16.18	49.48	30.0325
	STMN1 S38	428.6525	317.9225	581.68	371.975
	Perilipin 1 S174	61.585	49.1925	1674.508	1171.593
	FAM193A S1136	28.225	29.3575	46.44	31.035
	NFIB S264	78.3625	64.3625	93.8975	70.175
	NOP56 S554	35.26	31.2775	107.065	78.8075
	KMT2D S2299	20.4975	15.005	34.6125	22.285
	NFIL3 S353	15.6975	17.8175	26.375	20.5575
	RPS6KA3 S415	45.1175	47.4875	79.9325	63.5
	CHD8 S2040	13.6325	15.3025	29.7975	19.275
	QARS1 S70	139.1	146.2525	204.23	158.69
	NELFA T277	12.4225	17.18	89.215	52.1025
	ARAF T186	9.7425	8.0275	37.885	21.0325
	PALD1 S347	28.81	27.975	52.44	30.875
	Q922M7 S193	17.96	15.89	32.855	19.315
	FAM114A2 T121	63.2	51.31	104.5125	64.045
	SETD1A T1174/S1178	5.220022	6.229396	22.59295	15.27223
	THRAP3 S238/243	31.52507	22.28281	68.30888	36.47694

Phosphorylation levels of perilipin 1 and HSL

Phosphorylation level	37 ºC + Vehicle	27 ºC + Vehicle	37 ºC + NE	27 ºC + NE
HSL S34	9.1325	9.745	10.91	12.96
HSL S559	10.525	11.955	11.6575	14.165
HSL S597	27.895	27.4375	14.8975	19.61
HSL S651	71.0175	67.8925	73.9125	65.3725
HSL T752	27.135	25.965	85.3375	141.93
Perilipin 1 S384	254.175	330.355	2386.205	2683.3225
Perilipin 1 S460	31.2825	26.315	128.9275	106.005

Table S2. gRNA target sequences and ssDNA donor sequences used for the generation of perilipin 1^{FY4A} knock-in mice

bioRxiv preprint doi: https://doi.org/10.64898/2026.10.22.760470; this version posted October 27, 2026. The copyright holder for this preprint (which was not certified by peer review) is the author/funder, who has granted bioRxiv a license to display the preprint in perpetuity. It is made available under a CC-BY 4.0 International license.

sgRNA-A	ACCGAGGCGTCAGCGCGTCC
sgRNA-B	ACCTTCCCGTGGGGCGAGCA
ssDNA	CGCGCCCGGGCCAGCCCCGCGGCAGCTTGCGCAGCGTGCGGGGTCTCAGCGCGCCCTCCTGCCCCGGCCTGGACGACAAAACaGAGGCcTCcGCcCGcCCCGGCgcCCTGGCTATGCCCAGAGAGAAGCCTGCGCGCAGAGTCAGCGACAGCgcggcCCGGCCCAGCGTCATGGAGCCCATCCTGGGCCGCGCGCAGgccAGCCAGCTGCGCAAGAAGAGCTGAGCAGACTGCGCggCTGCTCGCCCCACGGGAAGGTCGCTTCTCGTCAAGGGCCTTTCCTTTAGGGTGGCCTGAGCCACGACCCCACGATGCTCTC
Genotyping forward primer	AGTGATCCCTAACAAGGGTGCC
Genotyping reverse primer	ACCTTCCCGTGGGGCGAGCA

Table S3. RT-qPCR primer sequences

bioRxiv preprint doi: https://doi.org/10.64898/2026.10.22.760470; this version posted October 27, 2026. The copyright holder for this preprint (which was not certified by peer review) is the author/funder, who has granted bioRxiv a license to display the preprint in perpetuity. It is made available under a CC-BY 4.0 International license.

species	Gene	Forward primer	Reverse primer
Mouse	*Actb*	GTGACGTTGACATCCGTAAAGA	GCCGGACTCATCGTACTCC
	Ucp1	AGGCTTCCAGTACCATTAGGT	CTGAGTGAGGCAAAGCTGATTT
	Ppargc1a	CCCTGCCATTGTTAAGACC	TGCTGCTGTTCCTGTTTTC
	Elovl3	TTCTCACGCGGGTTAAAAATGG	GGCCAACAACGATGAGCAAC
	Dio2	ATGGGACTCCTCAGCGTAGAC	ACTCTCCGCGAGTGGACTT
	Cox7a1	GCTCTGGTCCGGTCTTTTAGC	GTACTGGGAGGTCATTGTCGG

gRNA target sequences for generation of *Trpm8* and *Plin1* knockout adipocytes

sg*Trpm8*-A	ACCAATGATCGCCGCTGGG
sg*Trpm8*-B	GCACAGTCTAAAGGTACGC
sg*Trpm8*-C	TTCACCAATGATCGCCGCT
sg*Plin1*-A	TACCCGCCTTCGAAGCGCC
sg*Plin1*-B	CATGGAGCCGGTGGTCCGT
SgCon	GAGGCTAAGCGTCGCAAGTT
